# Molecular Structure and Properties of Resistant Dextrins from Potato Starch Prepared by Microwave Heating

**DOI:** 10.3390/ijms252011202

**Published:** 2024-10-18

**Authors:** Kamila Kapusniak, Malwina Wojcik, Justyna Rosicka-Kaczmarek, Karolina Miśkiewicz, Barbara Pacholczyk-Sienicka, Leslaw Juszczak

**Affiliations:** 1 Department of Dietetics and Food Studies, Faculty of Science and Technology, Jan Dlugosz University in Czestochowa, Armii Krajowej 13/15, 42-200 Czestochowa, Poland; m.wojcik@ujd.edu.pl; 2 Institute of Food Technology and Analysis, Faculty of Biotechnology and Food Sciences, Lodz University of Technology, Stefanowskiego 2/22, 90-537 Lodz, Poland; justyna.rosicka-kaczmarek@p.lodz.pl (J.R.-K.); karolina.miskiewicz@p.lodz.pl (K.M.); 3 Institute of Organic Chemistry, Faculty of Chemistry, Lodz University of Technology, Zeromskiego 116, 90-924 Lodz, Poland; barbara.pacholczyk-sienicka@p.lodz.pl; 4 Department of Food Analysis and Evaluation of Food Quality, Faculty of Food Technology, University of Agriculture in Krakow, Balicka 122, 30-149 Krakow, Poland; leslaw.juszczak@urk.edu.pl

**Keywords:** resistant dextrin, dietary fiber, potato starch, microwave heating

## Abstract

The dextrinization of potato starch was performed using a sophisticated single-mode microwave reactor with temperature and pressure control using 10 cycles of heating with stirring between cycles. Microwave power from 150 to 250 W, a cycle time from 15 to 25 s, and two types of vessels with different internal diameters (12 and 24 mm) and therefore different thicknesses of the heated starch layer were used in order to estimate the impact of vessel size used for microwave dextrinization. The characteristics of resistant dextrins (RD) including solubility in water, total dietary fiber (TDF) content, color parameters, the share of various glycosidic bonds, and pasting and rheological properties were carried out. The applied conditions allowed us to obtain RDs with water solubility up to 74% at 20 °C, as well as TDF content up to 47%, with a predominance of low-molecular-weight soluble fiber fraction, with increased content of non-starch glycosidic bonds, negligible viscosity, and a slightly beige color. The geometry of the reaction vessel influenced the properties of dextrins obtained under the same heating power, time, and repetition amounts. Among the conditions used, the most favorable conditions were heating 10 times for 20 s at 200 W in a 10 mL vessel and the least favorable were 15 s cycles.

## 1. Introduction

A high prevalence of obesity and its related chronic diseases is one of the main worldwide causes of mortality [1]. Although obesity does not lead to radical health complications in a short time, different health issues trigged by obesity or overweight may cause further deterioration of health in the long run, e.g., cardiovascular disease or micronutrient deficiencies, which can cause many further health complications [2]. In order to reduce this effect, it is necessary to change many lifestyle and eating habits, in particular limiting the consumption of simple sugars and increasing the content of fiber-rich products in one’s diet [3]. The most important factor seems to be the increased consumption of soluble dietary fiber that also has prebiotic properties, such as resistant dextrins—a well-known type of soluble dietary fiber [4,5] with prebiotic properties [6]. Resistant dextrins have been shown to have many health benefits, such as preventing obesity and diabetes and reducing inflammation via the reduction in food intake, the production of short-chain fatty acids (SCFA), and the influence of gut microbiota, cytokine production and macrophage activation [7,8,9].

Dextrinization is a process that leads to the creation of new, non-starch glycosidic bonds—the reason for enzymatic resistance and an influence on human health [10]. Depending on the selection of dextrinization conditions, as well as the botanical origin of starch, the final products may have different properties. One of the most important is very good solubility in water at a temperature of 20 °C, the lowest possible viscosity of solutions, the lightest possible color, and obviously the highest possible TDF content [11]. Dietary fiber products traditionally used in foods typically have much higher insoluble fiber content and therefore have different physiological benefits, primarily not providing the benefits of soluble, fermentable dietary fiber [12]. Moreover, some fiber preparations belong to the group of food additives, e.g., pectin [13]. RDs as a food ingredient may find wider use in food, following the clean label trend desired by modern consumers [14]. So far, the use of RDs has been described, among others, in dairy products [15], juices [16], cookies [17] and non-alcoholic beer [18].

RD have been obtained from starches of various botanical origins, such as potato, normal and waxy maize, cassava, rice, banana, yam, barley, and many other starch types [19]. Dextrinization is mainly performed using the convectional heating of starch acidified with hydrochloric [20,21,22] or acetic [23,24] acid or a combination of hydrochloric acid with an organic acid [25,26] as process catalysts. Importantly, the use of catalytic amounts of hydrochloric and citric acids for the dextrinization of starch does not introduce new chemical groups into the molecules, and it can be concluded that esterification does not occur [27]. It has been shown that RDs can be obtained by using either convectional or microwave heating, whether in a domestic microwave oven [28] or in a microwave reactor [29]. According to our knowledge, we are the only authors who have been performed dextrinization in a microwave reactor with temperature and pressure control, which is crucial in the case of microwave heating. Compared to conventional heating, microwave heating increases the rates of some chemical reactions by 10 to 1000 times [30]. Reactions carried out using microwave radiation are cleaner, more environmentally friendly, and cheaper in comparison to traditional heating. Microwave heating creates the possibility of carrying out new reactions that are not possible in the case of conventional heating [31]. Because of the rapid heating effect, microwaves cause the rearrangement of intramolecular structures of starch affecting gelatinization, solubility, swelling ability, pasting characteristics, dehydration, and rheological properties [32]. Based on the research of other authors [33], it can be hypothesized that the geometry of the vessel used in the microwave reactor to perform dextrinization may affect the properties of RDs.

The aim of this study was to check how the vessel geometry in which the starch was subjected to microwave dextrinization influences the properties of RDs. The possibility of preparing and examining the properties of dextrins obtained by reaction using heating in a CEM microwave reactor with temperature and pressure control was checked using two types of vessels—10 mL (internal diameter: 12 mm, height: 90 mm) and 35 mL (internal diameter: 24 mm, height: 90 mm) vessels.

## 2. Results and Discussion

### 2.1. Water Solubility of RDs

**Figure 1:**
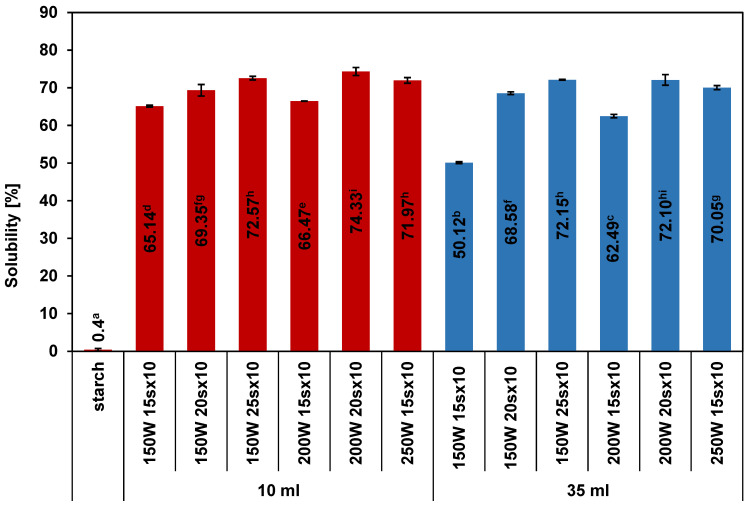
Solubility of potato starch and RDs prepared by microwave heating at 150, 200, and 250 W for 15, 20, and 25 s repeated 10 times in 10 mL (red) and 35 mL (blue) vessels. Different superscript lowercase letters (a, b, …) indicate significant differences (*p* < 0.05) between each RDs and starch.

The solubility in water at 20 °C of most of RDs was about 70%, except for the 150 W 15 s × 10 sample obtained in a 35 mL vessel, for which it was only about 50% (Figure 1). RDs obtained by heating at different power levels in 15 s cycles in 10 mL vessels were characterized by significantly higher solubility than RDs obtained in 35 mL vessels. Other samples showed similar solubility regardless of the type of vessel used. In both types of vessel, the solubility increased with the extension of the heating cycle duration at the same power and with the increase in the cycle power at the same time. The same result was obtained in previous studies [29]. The solubilities of the obtained RDs were lower those that of most pyrodextrins obtained from potato starch [26] and from starches of other botanical origins [34,35,36], but they were higher than almost all dextrins obtained as a result of dextrinization using a single-mode microwave reactor [26]. In the latter, a 35 mL vessel was used and only 5 g of dried starch was heated in continuous mode (lower solubility up to about 43%) and discontinuous mode (higher solubility up to 81%). The similar solubilities of the RDs obtained by discontinuous heating and those obtained when scaled up to 17.5 g, although still lower than the solubility of RDs obtained in 10 mL vessels, can be considered a favorable result.

### 2.2. Dietary Fiber Content of RDs

**Figure 2:**
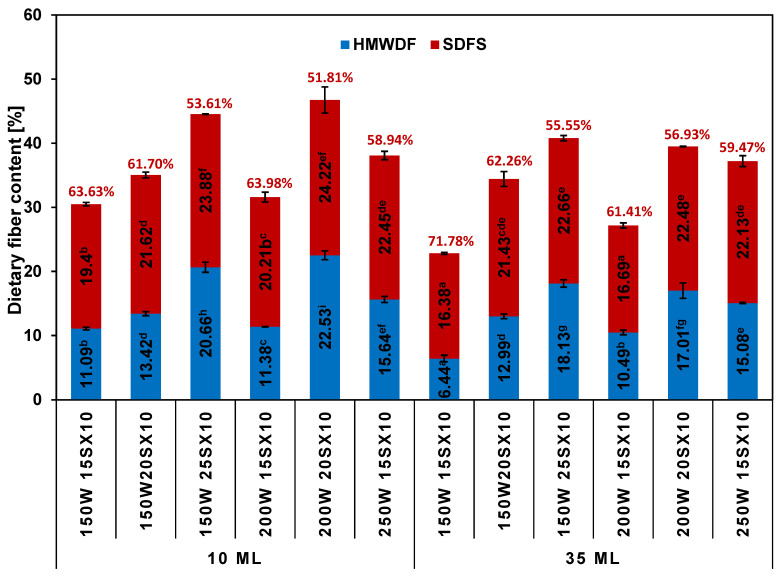
The dietary fiber contents of RDs prepared in 10 mL (**left**) and 35 mL (**right**) vessels by microwave-assisted heating. Different superscript lowercase letters (a, b, …) indicate significant differences (*p* < 0.05) between each parameter for each RDs. The red inscriptions are the percentage contents of the low-molecular fraction of the fiber (SDFS, always more than 50%).

TDF, as well as the share of low-molecular (SDFS) and high-molecular fractions (HMWDF) of RDs, varied depending on the dextrinization conditions used. The content of the HMWDF fraction in RDs obtained in 10 mL vessels ranged on average from 11.19 to 22.53%, while in 35 mL vessels, it ranged from 6.44 to 18.13%. In the case of the SDFS fraction, it ranged from 19.40 to 24.22% (in 10 mL vessels) and from 16.38 to 22.66% (in 35 mL vessels), respectively. In majority of the RDs, the TDF content, as well as the contents of their individual fractions for RDs obtained under the same conditions, was higher in 10 mL vessels. In each type of vessel, TDF increased with the extension of the heating cycle duration at the same power and with the increase in the cycle power at the same time. In all RDs, the low-molecular-weight fraction of fiber (determined by HPSEC) was dominant, ranging from 51.81 to 71.78% (Figure 2). The TDF contents of RDs were higher than for all pyrodextrins when they were determined by the same or a similar method [37,38,39]. Furthermore, the TDF contents of all RDs were higher than those of dextrins obtained in previous studies by continuous microwave heating and were the same or higher than the fiber contents of the best samples obtained by discontinuous heating [29]. The reason may be the thinner layer of heated starch in the smaller vessel (two times smaller internal diameter)—in the 10 mL vessel, the starch may have been affected more by microwaves than in the larger vessel.

### 2.3. Color Parameters (L* a* b*) of RDs

All RDs were visually characterized by a darker light beige color compared to native starch, which was confirmed by both a much lower value for the L* parameter and high ΔE values (from 11.51 to 20.84, Table 1). Compared to native potato starch, the RDs showed a significantly higher proportion of red color (a* value from 1.12 to 3.38 compared to 0.03 for starch) and yellow color (b* value from 10.73 to 16.12 compared to 1.84 for starch). It is well known that during the heating of starch in the presence of acid in elevated temperatures, Maillard reactions and caramelization products may be created [40]. For almost all color parameters, the values received for RDs obtained in 10 mL vessels indicated a higher level of starch modification than RDs obtained under the same conditions in 35 mL vessels. These RDs were characterized by lower L* values and higher values of other color parameters. In both types of vessel, the difference in color increased and the lightness decreased with the extension of the heating cycle duration at the same power and with the increase in the cycle power at the same time. The difference in color of RDs compared to native starch was lower than that for most yam-derived dextrins [35], similar or higher than that for cassava-derived dextrins [37] and Makal starch [41], and higher than the difference in color for dextrins obtained from waxy maize starch [34] and normal and waxy tapioca starch [42].

Compared to the previously conducted studies for samples obtained using a microwave reactor [29], as in the case of previous methods, the color parameters were the closest to the best samples obtained as a result of discontinuous heating. Additionally, for some samples, a higher degree of starch modification was observed than for all samples obtained in previous studies—higher values of ΔE, a*, and b* and lower values of L*. Among the previously mentioned studies, only in the case of cassava dextrins was dietary fiber content determined using the same method (AOAC 2009.01). However, the same tendency could be observed, namely that the darkest samples (the lowest L* and WI values) contained the most dietary fiber [34,37]. It can be assumed that carrying out the purification process in further research could not only allow us to obtain products with a more acceptable, lighter color but could also lead to a further increase in the enzyme-resistant fraction content without compromising other properties, i.e., without further reducing water solubility and increasing dextrin viscosity.

**Figure 3:**
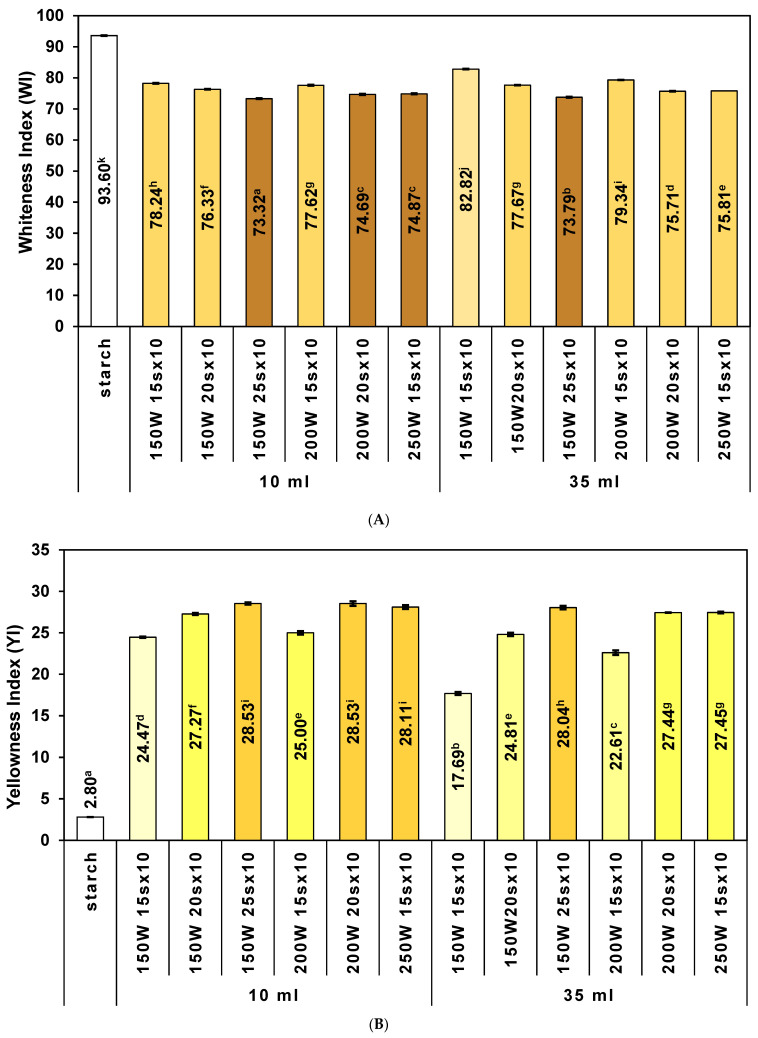
Whiteness index (**A**) and yellowness index (**B**) of potato starch and RDs prepared in 10 mL (**left**) and 35 mL (**right**) vessels by microwave-assisted heating. Different superscript lowercase letters (a, b, …) indicate significant differences (*p* < 0.05) between each parameter for each dextrin and starch.

In addition, whiteness and yellowness indexes for starch and RDs were calculated (Figure 3A,B, respectively). This seems to be a very useful and easy-to-interpret method of assessing the course of the dextrinization reaction and comparing the color of the obtained dextrins with the color of native starch. In the case of potato starch, which can be described as white, the difference in the color of the obtained RDs may seem greater than in the cases of starches of other botanical origin, like yam or cassava [35,37], which have higher b* values (yellow content). It turns out, however, that the percentage decrease in the obtained whiteness index values for RDs compared to potato starch ranged from 12 to 22% and was comparable to dextrins obtained from cassava starch, for which it ranged from 4 to 24% [37]. In both types of vessel, WI decreased with the extension of the heating cycle duration at the same power and with the increase in the cycle power at the same time.

The yellowness indices calculated for starch and RDs confirmed a significant increase in the share of yellow color in RDs and increased 6–10 times compared to potato starch. To the knowledge of the authors, the yellowness index has so far been determined only for products of other starch modifications and not for dextrinization products [43,44]. According to the authors, it can be a good tool for comparing the colors of dextrins obtained in different studies. YI increased with the extension of the heating cycle duration at the same power and with the increase in the cycle power at the same time in both types of vessels. The same tendency was observed for the lightness of samples (L*), and the reverse was observed for the whiteness indexes (WI).

### 2.4. Glycosidic Linkage Compositions Determined by NMR Spectroscopy of RDs

The types and proportions of glycoside linkages in the potato starch and RDs samples were analyzed using NMR spectroscopy (Figure S1). The type of glycosidic bond is responsible for enzymatic resistance and potential health effects; therefore, knowledge about possible changes in the structures of polysaccharides, which can be observed using NMR spectra, is very important. The ^1^H-NMR spectra of RDs showed anomeric hydrogen signals at 5.45, 5.37, 5.22, 5.12, 4.95, 4.77, 4.63, 4.61, and 4.5 ppm (Figure S2). The chemical shift positions of the signals from anomeric protons were in agreement with those previously reported [34]. Additionally, the degree of branching, average chain length, and degree of polymerization of potato starch and RDs were calculated (Table 2). The glycosidic linkages in RDs were significantly different from those in native potato starch. After dextrinization, the degree of α-1,4 linkages significantly decreased, degree of α-1,6 linkages increased, and new glycosyl linkages, including β-1,6, α-1,2, β-1,2, and β-1,4 linkages, were formed. RDs obtained in 10 mL vessels were characterized by a more favorable or similar share of glycosidic bonds in relation to RDs obtained in 35 mL vessels: they had a statistically significant lower or similar contents of 1,4-glycosidic bonds, higher or similar contents of the new α- and β-1,2- and α- and β-1,6-glycosidic bonds, and higher degrees of branching. As observed by other authors [34], as a result of dextrinization, the number of α-1,4-glycosidic bonds decreased to almost 70%, with a simultaneous low content of reducing ends (<3% of total α- and β-reducing ends). The degree of branching determined for potato starch was 1.52% and significantly increased to the values from 10% to almost 15%. These results were in line with results obtained by Han et al. [45] for dextrins prepared from waxy maize starch and lower than those obtained for dextrins prepared from cassava starch [39]. The most abundant branch type was the α-1,6 linkage, which increased from 1.52% to 5.68%. The most abundant non-starch glycosidic linkage types were β-1,6 (max. 4.93%) and α-1,2 (max. 2.54%). Additionally, lower contents of β-1,2 and β-1,4 linkages were observed, representing up to 1.28% and 0.48%, respectively. The calculated average chain lengths (3.53–4.61) and degrees of polymerization (10.79–13.23) of RDs were similar to dextrins obtained from waxy tapioca starch [38]. On the other hand, for dextrins prepared from waxy maize starch, [34] obtained similar CLn values but higher values of DP.

Transglucosylation occurring as a result of the applied process was confirmed by using NMR. The presence of new glycosidic bonds absent in native starch was confirmed. The appearance of new, indigestible glycosidic bonds in the human digestive system was the cause of the observed increased resistance to enzymatic digestion (high dietary fiber content).

NMR spectroscopy is a versatile and reliable technique for determining the changes in the molecular structure of potato starch during dextrinization. Through NMR, not only can new glycosidic linkages be easily identified, but quantitative information can also be obtained. It provides valuable data on the average degree of branching, average degree of polymerization, and average chain length in a single experiment. Moreover, NMR spectroscopy is a non-destructive technique, meaning that the sample is not altered or destroyed during analysis, allowing for multiple measurements to be taken on the same sample if needed.

### 2.5. Pasting Properties of RDs

**Figure 4:**
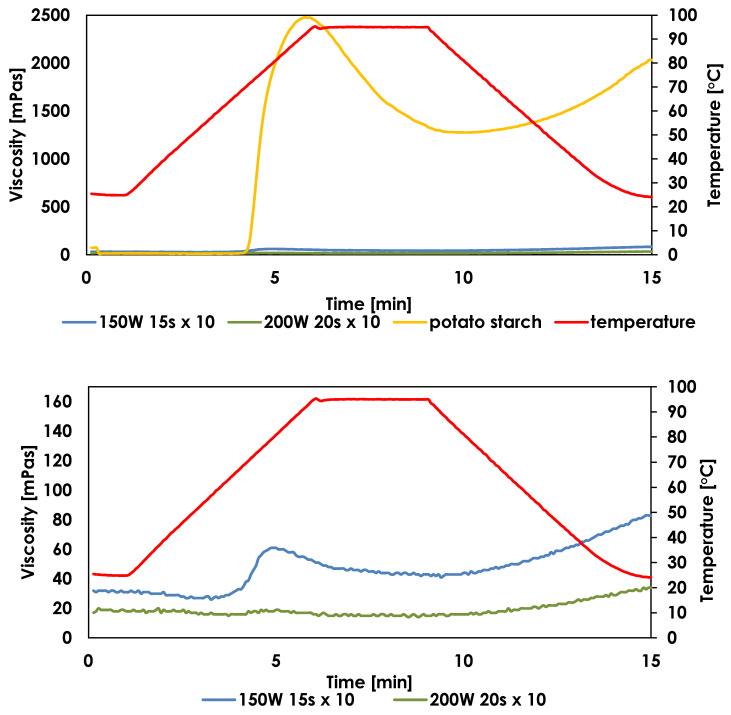
A comparison of pasting characteristics of potato starch (5%) and RDs (20%) obtained after the exposure of potato starch to microwave heating in 10 mL and 35 mL vessels (**up**), and a comparison of the pasting curves of the samples with the highest and the lowest pasting parameters of all RDs (**down**, temperature profile in red).

As is shown in Figure 4, pasting profiles of RDs are nearly flat in comparison to the pasting curve of potato starch. For only one, least modified sample, peak viscosity reached 60 mPas and final viscosity was 42 mPas, but these values were significantly higher than those for the rest of the samples and still far below values obtained for potato starch paste (Table S1). These findings are in line with previous results for dextrins obtained by microwave heating in a multimode oven [28], where the highest value of PV from dextrins was 31 mPas and that of FV was 48 mPas. Moreover, Li et al. [4] also observed that the viscosity of dextrins is nearly negligible in comparison with that of native starch, indicating that the dextrin solutions are close to a Newtonian low-viscosity fluid. From the point of view of potential applications, all samples (except sample 150 W 15 s × 10 obtained in a 35 mL vessel) were characterized by such low viscosity parameters that it is possible to add them to a wide range of products without increasing their viscosity. The dextrinization reaction determined that there was no significant decrease in viscosity during heating (RD was thermally stable due to the low breakdown value) and there was no increase in viscosity during cooling (no high setback values were observed, which could indicate a high tendency toward retrogradation).

### 2.6. Rheological Properties of RDs

**Figure 5:**
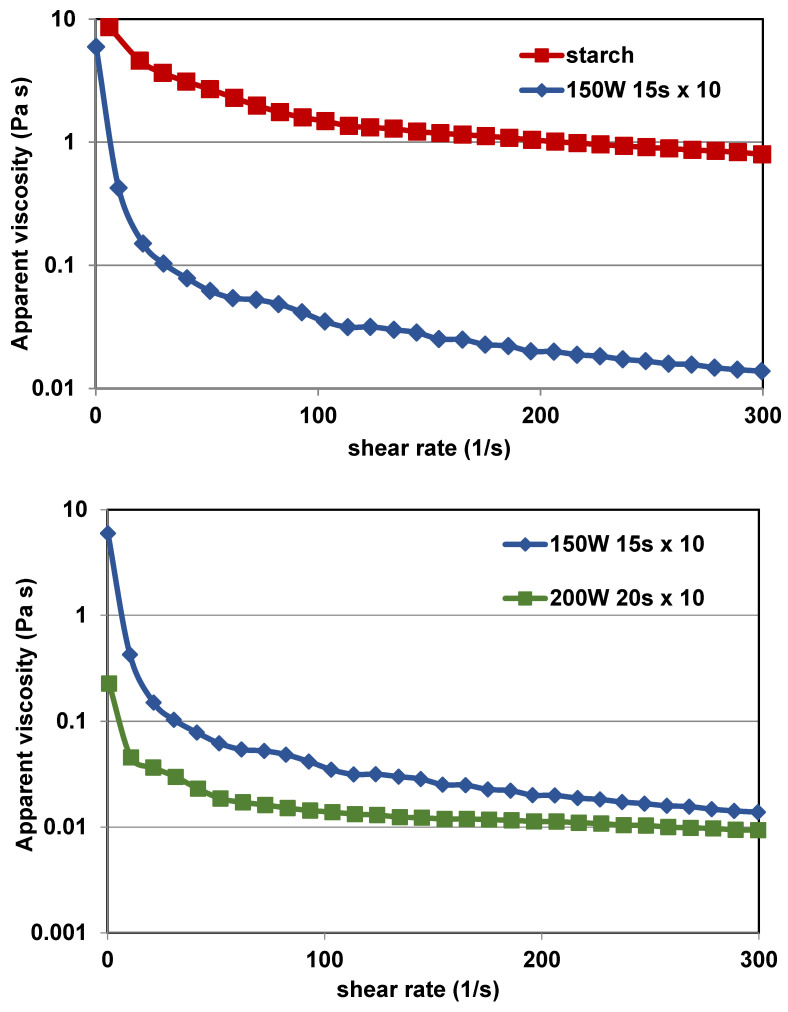
The viscosity curves of native potato starch and RDs with the highest viscosity (**up**) and a comparison of viscosity curves of RDs with the highest and the lowest viscosities (**down**).

All RDs samples displayed weak shear-thinning behavior. It was observed that the apparent viscosities of RDs and potato starch were significantly different throughout the measurement. Native starch paste showed a much higher apparent viscosity than RDs solutions (Figure 5), which resulted from the lower molecular weights of amylose and amylopectin degradation products [4]. The apparent viscosity of RDs decreased with an increase in the shear rate and was constantly near 60 s^−1^, confirming the non-Newtonian flow of the samples.

The RDs samples were characterized by low viscosity, but it varied depending on the degree of degradation of amylose and amylopectin to compounds with lower molecular weights (Table S2). Samples with the highest solubilities were characterized by the lowest viscosities, which could be correlated with the highest degree of starch depolymerization. In most other studies, the authors report very low viscosities of dextrins, which is associated with thorough starch depolymerization and the low molecular weights of the resulting products. Pseudoplastic flow behavior was observed for maize starch dextrins [4], yam dextrins [35], and microwave-treated potato starch [31]. In a small number of papers, in particular for highly soluble dextrins, the authors observe the behavior of dextrins as Newtonian fluids, whose viscosity is independent of the shear rate [34]. Results can be related to formation of new branches and crosslinking of polysaccharides molecules [4].

## 3. Materials and Methods

### 3.1. Materials

Potato starch and analytical-grade reagents were purchased from Merck Life Science, Poznan, Poland; enzymatic kits for dietary fiber content determination were purchased from Megazyme, Wicklow, Ireland.

### 3.2. Preparation of RDs

RDs were prepared by using the following two-step procedure:

- The predrying of potato starch sprayed with catalytic amounts of hydrochloric and citric acid (to the final concentration of 0.1% of each acid on dry starch basis, dsb) at 110 °C for 2 h;
- The heating of predried starch in a microwave reactor (dextrinization step).

RDs were prepared according to a previously described procedure [29], except for the experiment using 2 types of vessels—10 mL and 35 mL—compatible with the Discover SP microwave reactor (CEM Corporation, Matthews, NC, USA), which were half full, and the following microwave heating conditions were used: microwave powers of 150, 200, and 250 W; heating times 15, 20, and 25 s; and 10 heating cycles with manual mixing between cycles to avoid non-uniform heating. Conditions were proposed based on screening tests conducted on a large group of samples, and only samples that did not caramelize in any of the vessels used were included in this study (Table 3).

**Figure 6:**
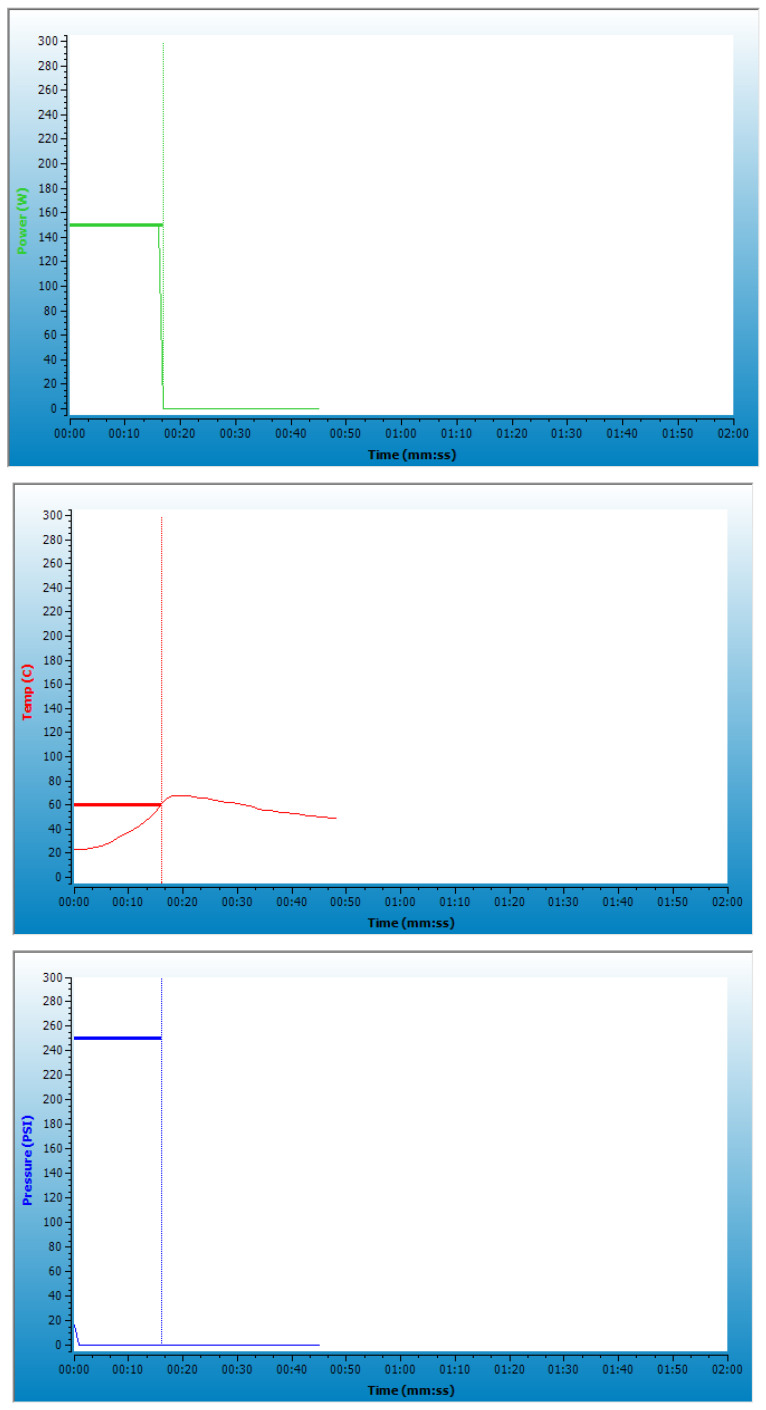
The power (**up**), temperaturę (**middle**), and pressure (**down**) conditions in a single heating cycle at 150 W for 15 s in a 10 mL vessel.

Samples were prepared by using the following Fix power program—heating at a set power for a specified time to a set control temperature (60 °C) with temperature, pressure, and power controlled as shown in Figure 6. Each sample was prepared in 4 repetitions and homogenized, and the average sample was taken for all further analyses.

### 3.3. Water Solubility

The water solubility of RDs was determined according to [28]. The sample (0.5 g) was suspended in 40 mL of distilled water and mixed for 30 min by using a magnetic stirrer. The suspension was then centrifuged at 21,381× *g* for 10 min, and 10 mL of supernatant was transferred into a weighing vessel of known weight. The weighing vessel was placed in an oven and heated to a constant weight at 130 °C. The water solubility of samples was calculated afterwards based on the weight of the residue after drying. The measurements were performed in triplicate.

### 3.4. Total Dietary Fiber Content (TDF)

TDF content was determined according to the official AOAC 2009.01 Method, allowing us to determine the content of high-molecular-weight dietary fiber (HMWDF), i.e., insoluble dietary fiber (IDF) and dietary fiber soluble in water, but precipitated in ethanol (SDFP), and dietary fiber soluble in water and not precipitated in ethanol (SDFS) [46]. In brief, samples were hydrolyzed with mixture of pancreatic α-amylase (porcine pancreatic) and amyloglucosidase (Aspergillus niger) for 16 h, then protease (Subtilisin A from Bacillus licheniformis) treatment was carried out for 30 min. HMWDF content was determined after the precipitation of SDFP with ethanol, filtration through crucible, washing, drying at 105 °C in an oven, and the determination of residue. The recovered filtrate was concentrated by evaporation under a vacuum at 60 °C to reach dryness and redissolved in 5 mL of deionized water. The deionization of the sample was performed using Amberlite and Ambersep resins. Eluate was concentrated by evaporation under vacuum at 60 °C to reach dryness and redissolved in 2 mL of deionized water, and it was analyzed with the HPSEC method [47] for SDFS content determination. The following conditions were used for the chromatographic analysis: column: PolySep-GFC-P 1000 LC, 300 × 7.8 mm; column temperature: 30 °C; eluent: 100% HPLC-grade water; flow rate: 0.4 cm^3^/min; analysis time: 40 min; injection volume: 0.01 cm^3^ (10 µL); detector RI: 40 °C. Glucose, maltose, and the maltooligosaccharides G3–G7 as molecular standards were used as references. The measurements were performed in duplicate.

### 3.5. Color Parameters (L* a* b*)

The color parameters of RDs were measured using a Chroma Meter CR-400 (Konica Minolta Sensing, Osaka, Japan) using the CIELAB system, D65 illuminant and 2° standard observer. L* (lightness), a* (red/green color), and b* (yellow/blue color) parameters were measured for native potato starch as controls. The color difference (ΔE) between starch and RDs was calculated with the following Equation (1):

(1) $$∆E=\sqrt{{\left(∆L^{*}\right)}^{2}+{\left({∆a}^{*}\right)}^{2}+{\left({∆b}^{*}\right)}^{2}}$$

where L*, a*, and b* were the mean values of the color parameters of RDs (measurements were performed 10 times for each sample). Additionally, the whiteness index (WI) and yellowness index (YI) were calculated with Equations (2) and (3) [44]:

(2) $$WI=100-\sqrt{{{\left(100-L^{*}\right)}^{2}+(a^{*})}^{2}+{\left(b^{*}\right)}^{2}}$$

(3) $$YI=\frac{142.86\times b^{*}}{L^{*}}$$

### 3.6. NMR Spectroscopy

All spectra were acquired using a Bruker Avance II Plus 16.4 T spectrometer (Bruker BioSpin, Ettlingen, Germany). The operating frequencies were 700 and 175 MHz for the ^1^H and ^13^C experiments. The instrument was equipped with a 5 mm Z-gradient broadband decoupling inverse probe. All experiments were performed (Figure S3) at 300 K. Samples (0.2 g) were dissolved in 0.7 mL of D_2_O (99.9% D) (DEUTERO GmbH, Kastellaun, Germany) with TSP (3-(trimethylsilyl)propionic-2,2,3,3-d4 acid sodium salt) (0.03% *v*/*v*) (Roth Industries GmbH & Co. KG, Dautphetal, Germany) and transferred to a high-quality 5 mm NMR tube.

The standard proton spectra were acquired with a calibrated 90° pulse for 128 scans collecting 64 K data points over a spectral width of 12 ppm. The repetition time of 6 s was matched to ensure complete magnetization recovery. The TSP peak at 0 ppm was used as a chemical shift standard.

All NMR spectra were manually phased, baseline corrected, and integrated using Topspin 3.2 (Bruker, Bremen, Germany). The peaks of anomeric protons were assigned to specific types of glycosidic bonds on the basis of a past study [34] and by comparison with the spectra of standards’ samples. Moreover, for precise chemical shift assignments, the ^1^H-^13^C HSQC NMR spectra were performed.

The spectral region from 4.4 to 5.7 ppm belonging to anomeric protons was integrated, and the relative amounts as percentages of the chemical structure corresponding to specific chemical shifts were calculated. Thus, the level of each type of glycosidic linkage was estimated as the integral of each specific peak divided by the summed integral of all anomeric protons. The degrees of branching, the average chain lengths, and degrees of polymerization of potato starch and RDs were calculated using Equations (4)–(6):

(4) $$DB=\frac{I_{4.50}+I_{4.61}+I_{4.77}+I_{4.95}+I_{5.12}}{I_{anomeric\;protons}}$$

(5) $$DP=\frac{I_{anomeric\;protons}}{I_{5.45}+I_{5.22}+I_{4.63}}$$

(6) $${CL}_{n}=\frac{I_{anomeric\;protons}}{I_{anomeric\;protons}-I_{5.34}}$$

### 3.7. Pasting Properties

The pasting properties of native potato starch and RDs were measured using Rapid Visco Analyzer (RVA 4500, Perten Instruments, Macquarie Park, Australia). The temperature profile previously described was used [28]: maintaining at 25 °C for 1 min, heating to 95 °C for 5 min, holding at 95 °C for 3 min, cooling to 25 °C for 5 min, and finally maintaining at 25 °C for 1 min (15 min in total). Measurements were carried out at a starch concentration of 5% and using RDs of 20%. Samples were mixed for the first 10 s at 960 rpm, then for the rest of the measurement at 160 rpm. For all samples, pasting characteristics were determined in duplicate and the following parameters were determined: peak viscosity (PV), hot paste viscosity (HPV), breakdown (BD = PV − HPV), final viscosity (FV), and setback (SB = FV − HPV).

### 3.8. Rheological Properties

The viscosity curves of the tested solutions were determined using a MARS II Rheometer (Thermo Fisher Scientific, Waltham, MA, USA) controlled by the RheoWin v 3.0 program (Thermo Fisher Scientific, USA) and equipped with a cone/plate measuring system (diameter 60 mm, angle 1°, measuring gap 0.052 mm). Starch samples were prepared by pasting their suspensions (5% *w*/*w*) in the RVA analyzer at a temperature of 95 °C, and after cooling to 50 °C, the obtained pastes were transferred to the measuring element of the rheometer and thermostated at the measurement temperature. RDs samples were prepared by heating their suspensions (20% *w*/*w*) with constant stirring at 50 °C for 2 h. Viscosity curves were determined at a temperature of 25 °C in the shear rate range from 1 to 300 s^−1^. The obtained curves were described with a power-law model:

(7) $$\eta_{ap.}=K\cdot {\dot{\gamma }}^{n-1}$$

where *η_ap_*.—apparent viscosity (Pa·s), *K*—consistency factor (Pa·s^n^), *γ*—shear rate, and *n*—flow index (-).

### 3.9. Statistical Analysis

The results were subjected to statistical analysis using Statistica 13.3 software (StatSoft, Tulsa, OK, USA). A completely randomized design was applied for all of the experiments. Analysis of variance was performed. Mean comparison was performed using Duncan’s new multiple range test (*p* < 0.05). The data were expressed as the mean values ± standard deviations.

## 4. Conclusions

The use of discontinuous microwave heating with mixing between cycles in a single-mode microwave reactor with temperature and pressure control allowed for the dextrinization of potato starch in a uniform manner. The applied conditions allowed us to obtain RDs with desired properties: high enzyme-resistant fraction content, high solubility in water, and low viscosity. It can be concluded that the geometry of the reaction vessel in which the sample is subjected to microwave heating influences the properties of the product. Conditions that were used in this study were suitable both for the use of half-size-full 35 mL and 10 mL vessels, but each time the use of smaller vessels allowed us to obtain more favorable results. Considering that the samples were not subjected to any purification at this stage of the research (e.g., with the use of membranes or activated carbon), they were characterized by a very high total dietary fiber content. However, achieving high resistance with the high solubility of samples in water at 20 °C at the same time remains a challenge.

## Figures and Tables

**Table 1:** Color parameters of potato starch and RDs obtained after exposure of potato starch to microwave heating in 10 mL and 35 mL vessels.

Sample	L*	a*	b*	ΔE
potato starch	93.87 ^k^	0.03 ^a^	1.84 ^a^	-
10 mL				
150 W 15 s ×10	83.76 ± 0.08 ^g^	2.04 ± 0.03 ^c^	14.35 ± 0.04 ^c^	16.20 ± 0.05 ^b^
150 W 20 s ×10	82.49 ± 0.08 ^e^	2.43 ± 0.05 ^e^	15.75 ± 0.06 ^hi^	18.12 ± 0.09 ^d^
150 W 25 s ×10	78.72 ± 0.13 ^a^	3.38 ± 0.02 ^h^	15.72 ± 0.09 ^ghi^	20.82 ± 0.07 ^i^
200 W 15 s ×10	83.13 ± 0.07 ^f^	2.16 ± 0.04 ^d^	14.54 ± 0.11 ^d^	16.77 ± 0.11 ^c^
200 W 20 s ×10	80.73 ± 0.15 ^c^	3.00 ± 0.04 ^g^	16.12 ± 0.09 ^k^	19.63 ± 0.13 ^g^
250 W 15 s ×10	80.75 ± 0.17 ^c^	2.92 ± 0.07 ^g^	15.89 ± 0.11 ^j^	19.43 ± 0.17 ^g^
35 mL				
150 W 15 s ×10	86.62 ± 0.11 ^i^	1.12 ± 0.03 ^b^	10.73 ± 0.10 ^b^	11.51 ± 0.13 ^a^
150 W 20 s ×10	83.11 ± 0.10 ^f^	2.15 ± 0.03 ^d^	14.74 ± 0.07 ^e^	16.66 ± 0.07 ^c^
150 W 25 s ×10	79.17 ± 0.09 ^b^	3.36 ± 0.02 ^h^	15.54 ± 0.13 ^g^	20.36 ± 0.04 ^h^
200 W 15 s ×10	84.60 ± 0.19 ^h^	2.18 ± 0.02 ^d^	14.43 ± 0.12 ^cd^	16.70 ± 0.08 ^c^
200 W 20 s ×10	82.36 ± 0.08 ^e^	2.49 ± 0.02 ^e^	15.28 ± 0.03 ^f^	18.27 ± 0.03 ^e^
250 W 15 s ×10	81.80 ± 0.08 ^d^	2.66 ± 0.04 ^f^	15.72 ± 0.04 ^i^	18.57 ± 0.07 ^f^

Different superscript lowercase letters (a, b, …) in the same column indicate significant differences (*p* < 0.05) between each parameter for each dextrin and starch.

**Table 2:** ^1^H chemical shifts of anomeric protons, proportions of the corresponding molecular structures, and degrees of branching (DB), average chain lengths (CLn), and degrees of polymerization (DP) of potato starch and RDs. Different superscript lowercase letters (a, b, …) indicate significant differences (*p* < 0.05) between each parameter for each dextrin and starch.

Sample	α(1-4) 5.37 ppm	α(1-6) 4.95 ppm	α(1-2) 5.12 ppm	β(1-4) 4.77 ppm	β(1-6) 4.50 ppm	β(1-2) 4.61 ppm	β(1-6) Anhydro 5.45 ppm	α-glc 5.22 ppm	β-glc 4.63 ppm	DB	CLn	DP
potato starch	92.44 ± 0.01 ^f^	1.52 ± 0.01 ^a^	0.00 ± 0.00 ^a^	0.00 ± 0.00 ^a^	0.00 ± 0.00 ^a^	0.00 ± 0.00 ^a^	0.00 ± 0.00 ^a^	1.60 ± 0.01 ^f^	1.60 ± 0.00 ^e^	1.52 ± 0.01 ^a^	13.22 ± 0.01 ^g^	31.30 ± 0.07 ^f^
10 mL												
150 W 15 s ×10	76.42 ± 0.54 ^d^	4.50 ± 0.28 ^c^	2.15 ± 0.07 ^b^	0.47 ± 0.04 ^cd^	3.66 ± 0.20 ^c^	0.93 ± 0.04 ^c^	6.00 ± 0.13 ^c^	0.97 ± 0.05 ^de^	1.10 ± 0.00 ^a^	11.71 ± 0.56 ^c^	4.24 ± 0.10 ^e^	13.23 ± 0.13 ^d^
150 W 20 s ×10	74.62 ± 0.11 ^c^	4.68 ± 0.18 ^cd^	2.42 ± 0.12 ^c^	0.38 ± 0.03 ^b^	3.93 ± 0.11 ^c^	1.02 ± 0.03 ^d^	6.67 ± 0.18 ^de^	0.99 ± 0.02 ^e^	1.35 ± 0.07 ^cd^	12.42 ± 0.40 ^cde^	3.94 ± 0.02 ^c^	11.11 ± 0.34 ^ab^
150 W 25 s ×10	73.50 ± 0.42 ^b^	5.20 ± 0.14 ^e^	2.25 ± 0.07 ^bc^	0.42 ± 0.03 ^bcd^	4.50 ± 0.28 ^d^	1.20 ± 0.00 ^e^	6.50 ± 0.28 ^d^	0.89 ± 0.02 ^c^	1.20 ± 0.00 ^b^	13.57 ± 0.47 ^fg^	3.77 ± 0.06 ^b^	11.65 ± 0.36 ^b^
200 W 15 s ×10	76.53 ± 0.11 ^d^	4.56 ± 0.21 ^c^	2.14 ± 0.09 ^b^	0.42 ± 0.02 ^bc^	3.74 ± 0.08 ^c^	0.98 ± 0.11 ^cd^	6.15 ± 0.07 ^cd^	0.81 ± 0.01 ^a^	1.15 ± 0.07 ^ab^	11.82 ± 0.25 ^c^	4.26 ± 0.02 ^e^	12.34 ± 0.20 ^cd^
200 W 20 s ×10	71.76 ± 0.22 ^a^	5.68 ± 0.18 ^f^	2.54 ± 0.08 ^cd^	0.39 ± 0.02 ^b^	4.93 ± 0.11 ^e^	1.28 ± 0.03 ^f^	7.10 ± 0.00 ^f^	0.82 ± 0.03 ^ab^	1.35 ± 0.07 ^cd^	14.81 ± 0.42 ^h^	3.54 ± 0.03 ^a^	10.79 ± 0.05 ^a^
250 W 15 s ×10	74.94 ± 0.05 ^c^	4.88 ± 0.03 ^d^	2.34 ± 0.09 ^c^	0.41 ± 0.01 ^b^	4.28 ± 0.17 ^d^	1.07 ± 0.04 ^d^	6.40 ± 0.15 ^d^	0.87 ± 0.04 ^bc^	1.23 ± 0.04 ^bc^	12.97 ± 0.33 ^ef^	3.99 ± 0.01 ^d^	11.78 ± 0.22 ^b^
35 mL												
150 W 15 s ×10	78.21 ± 0.01 ^e^	3.86 ± 0.21 ^b^	2.07 ± 0.05 ^b^	0.48 ± 0.04 ^c^	2.98 ± 0.17 ^b^	0.77 ± 0.10 ^bc^	5.55 ± 0.07 ^b^	0.91 ± 0.01 ^cd^	1.11 ± 0.13 ^ab^	10.15 ± 0.36 ^b^	4.59 ± 0.00 ^f^	13.23 ± 0.10 ^e^
150 W 20 s ×10	74.95 ± 0.36 ^c^	4.80 ± 0.14 ^a^	2.15 ± 0.07 ^b^	0.47 ± 0.04 ^c^	3.97 ± 0.09 ^c^	1.03 ± 0.04 ^d^	6.05 ± 0.07 ^c^	0.95 ± 0.07 ^cde^	1.20 ± 0.00 ^b^	12.41 ± 0.13 ^d^	3.99 ± 0.06 ^d^	12.20 ± 0.00 ^c^
150 W 25 s ×10	72.50 ± 0.71 ^ab^	5.24 ± 0.37 ^def^	2.25 ± 0.07 ^bc^	0.48 ± 0.03 ^c^	4.40 ± 0.14 ^d^	1.20 ± 0.00 ^e^	6.45 ± 0.21 ^de^	0.89 ± 0.02 ^c^	1.30 ± 0.14 ^bcd^	13.57 ± 0.62 ^fg^	3.64 ± 0.09 ^ab^	11.58 ± 0.07 ^b^
200 W 15 s ×10	78.32 ± 0.12 ^e^	3.97 ± 0.05 ^b^	2.10 ± 0.00 ^b^	0.47 ± 0.02 ^d^	3.00 ± 0.07 ^b^	0.74 ± 0.00 ^b^	5.72 ± 0.37 ^bc^	0.90 ± 0.04 ^cd^	1.19 ± 0.02 ^b^	10.27 ± 0.14 ^b^	4.61 ± 0.03 ^f^	12.82 ± 0.57 ^cde^
200 W 20 s ×10	71.65 ± 1.06 ^a^	5.35 ± 0.07 ^e^	2.15 ± 0.07 ^b^	0.42 ± 0.03 ^bcd^	4.42 ± 0.11 ^d^	1.22 ± 0.02 ^e^	6.45 ± 0.21 ^de^	1.01 ± 0.01 ^e^	1.28 ± 0.03 ^c^	13.56 ± 0.21 ^g^	3.53 ± 0.13 ^a^	11.45 ± 0.25 ^b^
250 W 15 s ×10	75.13 ± 0.25 ^c^	4.90 ± 0.29 ^de^	2.17 ± 0.05 ^b^	0.41 ± 0.01 ^b^	3.97 ± 0.18 ^cd^	1.05 ± 0.07 ^d^	6.07 ± 0.05 ^c^	0.98 ± 0.04 ^de^	1.20 ± 0.00 ^b^	12.49 ± 0.45 ^cde^	4.02 ± 0.04 ^d^	12.14 ± 0.12 ^c^

**Table 3:** Microwave processing conditions of RDs with successful attempts **✓** and failed attempts **x**.

	150 W × 10		200 W × 10		250 W × 10	
Duration of Heating Cycle [s]	10 mL	35 mL	10 mL	35 mL	10 mL	35 mL
15	**✓**	**✓**	**✓**	**✓**	**✓**	**✓**
20	**✓**	**✓**	**✓**	** ✓ **	**x**	**x**
25	**✓**	**✓**	**x**	**x**	**x**	**x**

## Data Availability

All data are included in this article.

## Supplementary Materials

The supporting information can be downloaded at https://www.mdpi.com/article/10.3390/ijms252011202/s1.

## References

[1] García O.P., Long K.Z., Rosado J.L. (2009). Impact of Micronutrient Deficiencies on Obesity. Nutr. Rev..

[2] The GBD 2015 Obesity Collaborators (2017). Health Effects of Overweight and Obesity in 195 Countries over 25 Years. N. Engl. J. Med..

[3] Wilson A.S., Koller K.R., Ramaboli M.C., Nesengani L.T., Ocvirk S., Chen C., Flanagan C.A., Sapp F.R., Merritt Z.T., Bhatti F. (2020). Diet and the Human Gut Microbiome: An International Review. Dig. Dis. Sci..

[4] Li H., Ji J., Yang L., Lei N., Wang J., Sun B. (2020). Structural and Physicochemical Property Changes during Pyroconversion of Native Maize Starch. Carbohydr. Polym..

[5] Hobden M.R., Commane D.M., Guérin-Deremaux L., Wils D., Thabuis C., Martin-Morales A., Wolfram S., Dìaz A., Collins S., Morais I. (2021). Impact of Dietary Supplementation with Resistant Dextrin (NUTRIOSE^®^) on Satiety, Glycaemia, and Related Endpoints, in Healthy Adults. Eur. J. Nutr..

[6] Lefranc-Millot C., Guérin-Deremaux L., Wils D., Neut C., Miller L.E., Saniez-Degrave M.H. (2012). Impact of a resistant dextrin on intestinal ecology: How altering the digestive ecosystem with NUTRIOSE^®^, a soluble fibre with prebiotic properties, may be beneficial for health. J. Int. Med. Res..

[7] Aliasgharzadeh A., Dehghan P., Gargari B.P., Asghari-Jafarabadi M. (2015). Resistant Dextrin, as a Prebiotic, Improves Insulin Resistance and Inflammation in Women with Type 2 Diabetes: A Randomised Controlled Clinical Trial. Br. J. Nutr..

[8] Nazare J.A., Sauvinet V., Normand S., Guérin-Deremaux L., Guérin-Deremaux L., Gabert L., Wils D., Laville M. (2011). Impact of a Resistant Dextrin with a Prolonged Oxidation Pattern on Day-Long Ghrelin Profile. J. Am. Coll. Nutr..

[9] Mateo-Gallego R., Moreno-Indias I., Bea A.M., Sánchez-Alcoholado L., Fumanal A.J., Quesada-Molina M., Prieto-Martín A., Gutiérrez-Repiso C., Civeira F., Tinahones F.J. (2021). An Alcohol-Free Beer Enriched with Isomaltulose and a Resistant Dextrin Modulates Gut Microbiome in Subjects with Type 2 Diabetes Mellitus and Overweight or Obesity: A Pilot Study. Food Funct..

[10] Nunes F.M., Lopes E.S., Moreira A.S.P., Simões J., Coimbra M.A., Domingues R.M. (2016). Formation of Type 4 Resistant Starch and Maltodextrins from Amylose and Amylopectin upon Dry Heating: A Model Study. Carbohydr. Polym..

[11] Liu Z., Liu J., Ren L., Wu J., Chen S. (2022). Preparation of High-Quality Resistant Dextrin through Pyrodextrin by a Multienzyme Complex. Food Biosci..

[12] Weickert M.O., Pfeiffer A.F.H. (2018). Impact of Dietary Fiber Consumption on Insulin Resistance and the Prevention of Type 2 Diabetes. J. Nutr..

[13] Huang J.Y., Liao J.S., Qi J.R., Jiang W.X., Yang X.Q. (2021). Structural and Physicochemical Properties of Pectin-Rich Dietary Fiber Prepared from Citrus Peel. Food Hydrocoll..

[14] Aschemann-Witzel J., Varela P., Peschel A.O. (2019). Consumers’ Categorization of Food Ingredients: Do Consumers Perceive Them as ‘Clean Label’ Producers Expect? An Exploration with Projective Mapping. Food Qual. Prefer..

[15] Saeedyzadeh N., Zamindar N., Pezeshkzadeh M., Tahmourespour A. (2017). Evaluation of Yogurt-like Beverages Made of Potato Starch Waste and Grape Must. J. Food Meas. Charact..

[16] Miravet G., Alacid M., Obón J.M., Fernández-López J.A. (2016). Spray-Drying of Pomegranate Juice with Prebiotic Dietary Fibre. Int. J. Food Sci. Technol..

[17] Yu S., Dong K., Pora B.L.R., Hasjim J. (2022). The Roles of a Native Starch and a Resistant Dextrin in Texture Improvement and Low Glycemic Index of Biscuits. Processes.

[18] Lamiquiz-Moneo I., Pérez-Calahorra S., Gracia-Rubio I., Cebollada A., Bea A.M., Fumanal A., Ferrer-Mairal A., Prieto-Martín A., Sanz-Fernández M.L., Cenarro A. (2022). Effect of the Consumption of Alcohol-Free Beers with Different Carbohydrate Composition on Postprandial Metabolic Response. Nutrients.

[19] Zarski A., Kapusniak K., Ptak S., Rudlicka M., Coseri S., Kapusniak J. (2024). Functionalization Methods of Starch and Its Derivatives: From Old Limitations to New Possibilities. Polymers.

[20] Bai Y., Shi Y.C. (2016). Chemical Structures in Pyrodextrin Determined by Nuclear Magnetic Resonance Spectroscopy. Carbohydr. Polym..

[21] Olvera-Hernández V., Betancur-Ancona D., Chel-Guerrero L.A., Ble-Castillo J.L., Castellanos-Ruelas A.F. (2018). Morphological and Physicochemical Changes in Great Dwarf Banana (Musa Cavendish AAA) Starch Modified by Pyrodextrinization and Enzymatic Hydrolysis. Starch/Staerke.

[22] Mao H., Li J., Chen Z., Yan S., Li H., Wen Y., Wang J. (2021). Molecular Structure of Different Prepared Pyrodextrins and the Inhibitory Effects on Starch Retrogradation. Food Res. Int..

[23] Lin C.L., Lin J.H., Zeng H.M., Wu Y.H., Chang Y.H. (2018). Indigestible Pyrodextrins Prepared from Corn Starch in the Presence of Glacial Acetic Acid. Carbohydr. Polym..

[24] de Souza Oliveira E., Lovera M., Rios Pires V., da Silva Mendes F.R., Lima Peixoto Maia N.V., Viana Rodrigues J.P., Rocha Bastos M.d.S., Cheng H.N., Biswas A., de Azevedo Moreira R. (2022). Effect of Acid Catalyst on Pyroconversion of Breadfruit (Artocarpus Altilis) Starch: Physicochemical and Structural Properties. J. Food Process Preserv..

[25] Barczynska R., Slizewska K., Jochym K., Kapusniak J., Libudzisz Z. (2012). The Tartaric Acid-Modified Enzyme-Resistant Dextrin from Potato Starch as Potential Prebiotic. J. Funct. Foods.

[26] Kapusniak K., Wojcik M., Wrobel K., Rosicka-Kaczmarek J., Kapusniak J. (2021). Assessment of Physicochemical and Thermal Properties of Soluble Dextrin Fiber from Potato Starch for Use in Fruit Mousses. J. Sci. Food Agric..

[27] Wojcik M., Kapusniak K., Zarski A., Kapusniak J. (2024). Preparation and Characterization of Soluble Dextrin Fibre from Potato Starch Obtained on a Semi-Industrial Scale. Appl. Sci..

[28] Jochym K.K., Nebesny E. (2017). Enzyme-Resistant Dextrins from Potato Starch for Potential Application in the Beverage Industry. Carbohydr. Polym..

[29] Kapusniak K., Lubas K., Wojcik M., Rosicka-Kaczmarek J., Pavlyuk V., Kluziak K., Gonçalves I., Lopes J., Coimbra M.A., Kapusniak J. (2021). Effect of Continuous and Discontinuous Microwave-Assisted Heating on Starch-Derived Dietary Fiber Production. Molecules.

[30] Kumar A., Kuang Y., Liang Z., Sun X. (2020). Microwave Chemistry, Recent Advancements, and Eco-Friendly Microwave-Assisted Synthesis of Nanoarchitectures and Their Applications: A Review. Mater. Today Nano.

[31] Kumar Y., Singh L., Sharanagat V.S., Patel A., Kumar K. (2020). Effect of Microwave Treatment (Low Power and Varying Time) on Potato Starch: Microstructure, Thermo-Functional, Pasting and Rheological Properties. Int. J. Biol. Macromol..

[32] Fan D., Wang L., Zhang N., Xiong L., Huang L., Zhao J., Wang M., Zhang H. (2017). Full-Time Response of Starch Subjected to Microwave Heating. Sci. Rep..

[33] Panzarella B., Tompsett G.A., Yngvesson K.S., Conner W.C. (2007). Microwave Synthesis of Zeolites. 2. Effect of Vessel Size, Precursor Volume, and Irradiation Method. J. Phys. Chem. B.

[34] Chen J., Xiao J., Wang Z., Cheng H., Zhang Y., Lin B., Qin L., Bai Y. (2020). Effects of Reaction Condition on Glycosidic Linkage Structure, Physical–Chemical Properties and In Vitro Digestibility of Pyrodextrins Prepared from Native Waxy Maize Starch. Food Chem..

[35] Lovera M., de Castro G.M.C., da Rocha Pires N., do Socorro Rocha Bastos M., Holanda-Araújo M.L., Laurentin A., de Azevedo Moreira R., de Oliveira H.D. (2020). Pyrodextrinization of Yam (*Dioscorea* sp.) Starch Isolated from Tubers Grown in Brazil and Physicochemical Characterization of Yellow Pyrodextrins. Carbohydr. Polym..

[36] Chen W., Zhang T., Ma Q., Zhu Y., Shen R. (2022). Structure Characterization and Potential Probiotic Effects of Sorghum and Oat Resistant Dextrins. Foods.

[37] Trithavisup K., Krusong K., Tananuwong K. (2019). In-Depth Study of the Changes in Properties and Molecular Structure of Cassava Starch during Resistant Dextrin Preparation. Food Chem..

[38] Weil W., Weil R.C., Keawsompong S., Sriroth K., Seib P.A., Shi Y.C. (2021). Pyrodextrins from Waxy and Normal Tapioca Starches: Molecular Structure and In Vitro Digestibility. Carbohydr. Polym..

[39] Trithavisup K., Shi Y.C., Krusong K., Tananuwong K. (2022). Molecular Structure and Properties of Cassava-Based Resistant Maltodextrins. Food Chem..

[40] Zhen Y., Zhang T., Jiang B., Chen J. (2021). Purification and Characterization of Resistant Dextrin. Foods.

[41] Barbosa-Martín E., Sauri-Duch E., Chel-Guerrero L., Cuevas-Glory L., Moo-Huchin V., Betancur-Ancona D. (2024). Synthesis of Pyrodextrins and Enzymatically Resistant Maltodextrins from Makal (*Xanthosoma yucatenensis*) Starch. Food Technol. Biotechnol..

[42] Weil W., Weil R.C., Keawsompong S., Sriroth K., Seib P.A., Shi Y.C. (2020). Pyrodextrin from Waxy and Normal Tapioca Starches: Physicochemical Properties. Food Hydrocoll..

[43] Piloni R.V., Bordón M.G., Barrera G.N., Martínez M.L., Ribotta P.D. (2022). Porous Microparticles of Corn Starch as Bio-Carriers for Chia Oil. Foods.

[44] Zhai X., Li M., Zhang R., Wang W., Hou H. (2023). Extrusion-Blown Starch/PBAT Biodegradable Active Films Incorporated with High Retentions of Tea Polyphenols and the Release Kinetics into Food Simulants. Int. J. Biol. Macromol..

[45] Han X., Kang J., Bai Y., Xue M., Shi Y.C. (2018). Structure of Pyrodextrin in Relation to Its Retrogradation Properties. Food Chem..

[46] McCleary B.V., DeVries J.W., Rader J.I., Cohen G., Prosky L., Mugford D.C., Champ M., Okuma K. (2012). Determination of Insoluble, Soluble, and Total Dietary Fiber (CODEX Definition) by Enzymatic-Gravimetric Method and Liquid Chromatography: Collaborative Study. J. AOAC Int..

[47] Gruska R.M., Baryga A., Kunicka-Styczyńska A., Brzeziński S., Rosicka-Kaczmarek J., Miśkiewicz K., Sumińska T. (2022). Fresh and Stored Sugar Beet Roots as a Source of Various Types of Mono- and Oligosaccharides. Molecules.

