# Supplementary material for: Molecular Structure and Properties of Resistant Dextrins from Potato Starch Prepared by Microwave Heating
Source: Int J Mol Sci. 2024 Oct 18;25(20):11202. doi: 10.3390/ijms252011202 (PMC11508830; doi:10.3390/ijms252011202)
Supplement: Supplementary file 1 [file ijms-25-11202-s001.zip › ijms-3248495-supplementary.pdf]

Supplementary data

Table S1. Pasting parameters in Pas of potato starch and RDs obtained after exposure of potato starch to microwave heating in 10 ml and 35 ml vessels. Different superscript lowercase letters (a, b, …) in the same column indicate significant differences ($p < 0.05$) between each parameter for each RD and starch.

Sample	PV	HPV	BD = PV-HPV	FV	SB = FV-HPV
potato starch	$2705±91^{g}$	$578±18^{f}$	$2127±109^{g}$	$803±27^{g}$	$226±9^{g}$
**10 ml**					
150W 15s x10	$33±4^{de}$	$18±2^{ab}$	$15±2^{e}$	$36±3^{abc}$	$19±2^{ab}$
150W 20s x10	$27±2^{cd}$	$20±1^{bc}$	$7±2^{abcd}$	$48±1^{d}$	$28±1^{e}$
150W 25s x10	$22±1^{ab}$	$16±0^{a}$	$6±1^{abc}$	$35±1^{a}$	$19±1^{a}$
200W 15s x10	$34±1^{e}$	$25±1^{d}$	$10±1^{d}$	$54±1^{e}$	$30±1^{e}$
200W 20s x10	$21±0^{a}$	$16±0^{a}$	$5±0^{a}$	$36±0^{a}$	$20±0^{a}$
250W 15s x10	$25±1^{c}$	$18±1^{b}$	$7±0^{c}$	$40±1^{c}$	$22±0^{c}$
**35 ml**					
150W 15s x10	$60±1^{f}$	$41±1^{e}$	$20±1^{f}$	$83±1^{f}$	$42±0^{f}$
150W 20s x10	$29±0^{d}$	$22±1^{c}$	$8±1^{cd}$	$47±1^{d}$	$25±0^{d}$
150W 25s x10	$24±1^{bc}$	$18±0^{b}$	$6±1^{abc}$	$39±1^{bc}$	$21±1^{abc}$
200W 15s x10	$36±4^{e}$	$26±1^{d}$	$10±1^{d}$	$55±1^{e}$	$29±0^{e}$
200W 20s x10	$25±1^{c}$	$19±1^{b}$	$6±0^{b}$	$38±0^{b}$	$19±1^{a}$
250W 15s x10	$25±1^{c}$	$19±2^{bc}$	$6±2^{abcd}$	$43±2^{c}$	$24±1^{d}$

Table S2. Power-law model parameters describing the flow curves of native starch and RDs. Different superscript lowercase letters (a, b, …) in the same column indicate significant differences ($p < 0.05$) between each parameter for each dextrin and starch.

Sample	K [Pas^n]	n [-]	R^2
potato starch	$29.74±1.52^{g}$	$0.36±0.02^{d}$	0.9916
**10 ml**			
150W 15s x10	$1.59±0.27^{f}$	$0.12±0.03^{a}$	0.9827
150W 20s x10	$0.81±0.04^{d}$	$0.21±0.03^{b}$	0.9808
150W 25s x10	$0.43±0.03^{b}$	$0.31±0.03^{cd}$	0.9846
200W 15s x10	$1.10±0.15^{e}$	$0.15±0.02^{a}$	0.9887
200W 20s x10	$0.16±0.02^{a}$	$0.49±0.03^{e}$	0.9769
250W 15s x10	$0.51±0.01^{c}$	$0.28±0.03^{c}$	0.9801
**35 ml**			
150W 15s x10	$1.84±0.19^{f}$	$0.15±0.03^{ab}$	0.9904
150W 20s x10	$1.03±0.03^{e}$	$0.17±0.03^{ab}$	0.9816
150W 25s x10	$0.47±0.04^{bc}$	$0.31±0.02^{c}$	0.9750
200W 15s x10	$1.79±0.03^{f}$	$0.17±0.05^{ab}$	0.9827
200W 20s x10	$0.50±0.05^{bc}$	$0.30±0.03^{c}$	0.9757
250W 15s x10	$0.52±0.04^{c}$	$0.29±0.01^{c}$	0.9855

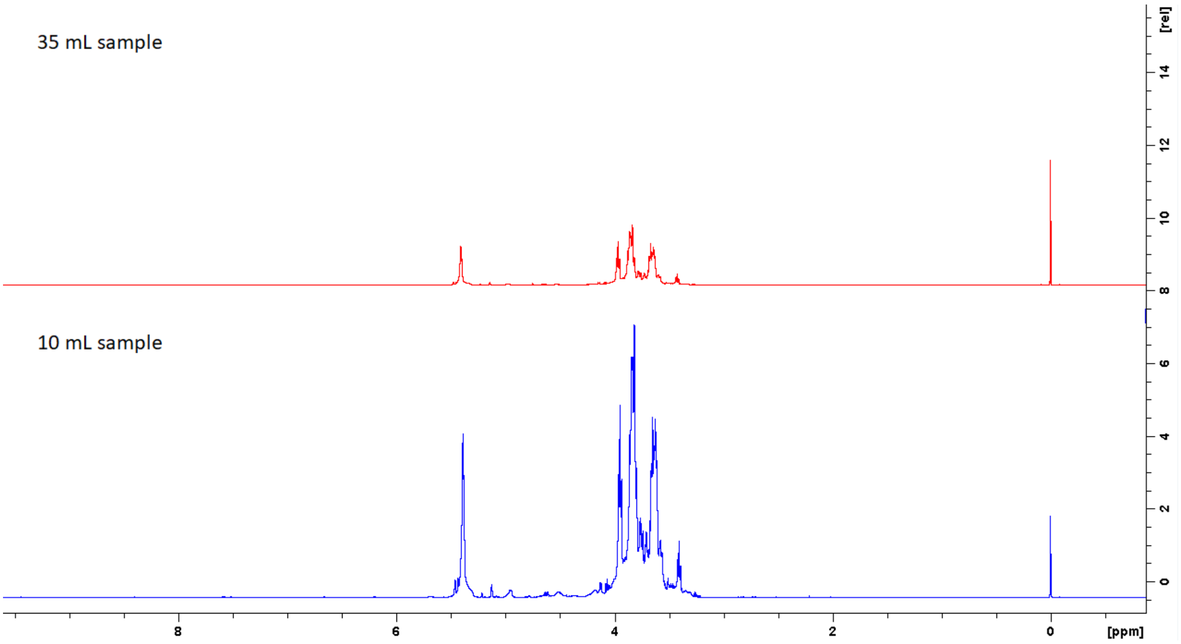

Figure S1. Representative ^{1}H-NMR spectra of two conditions (sample 10 mL and 35 mL).

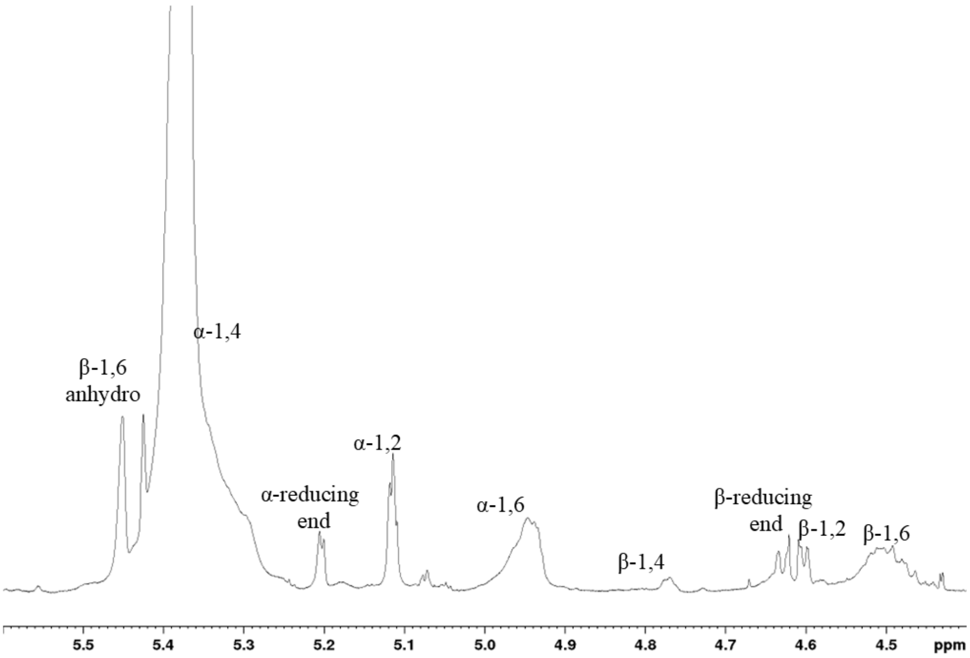

Figure S2. Representative ^{1}H-NMR spectrum of anomeric protons for 10 mL sample.

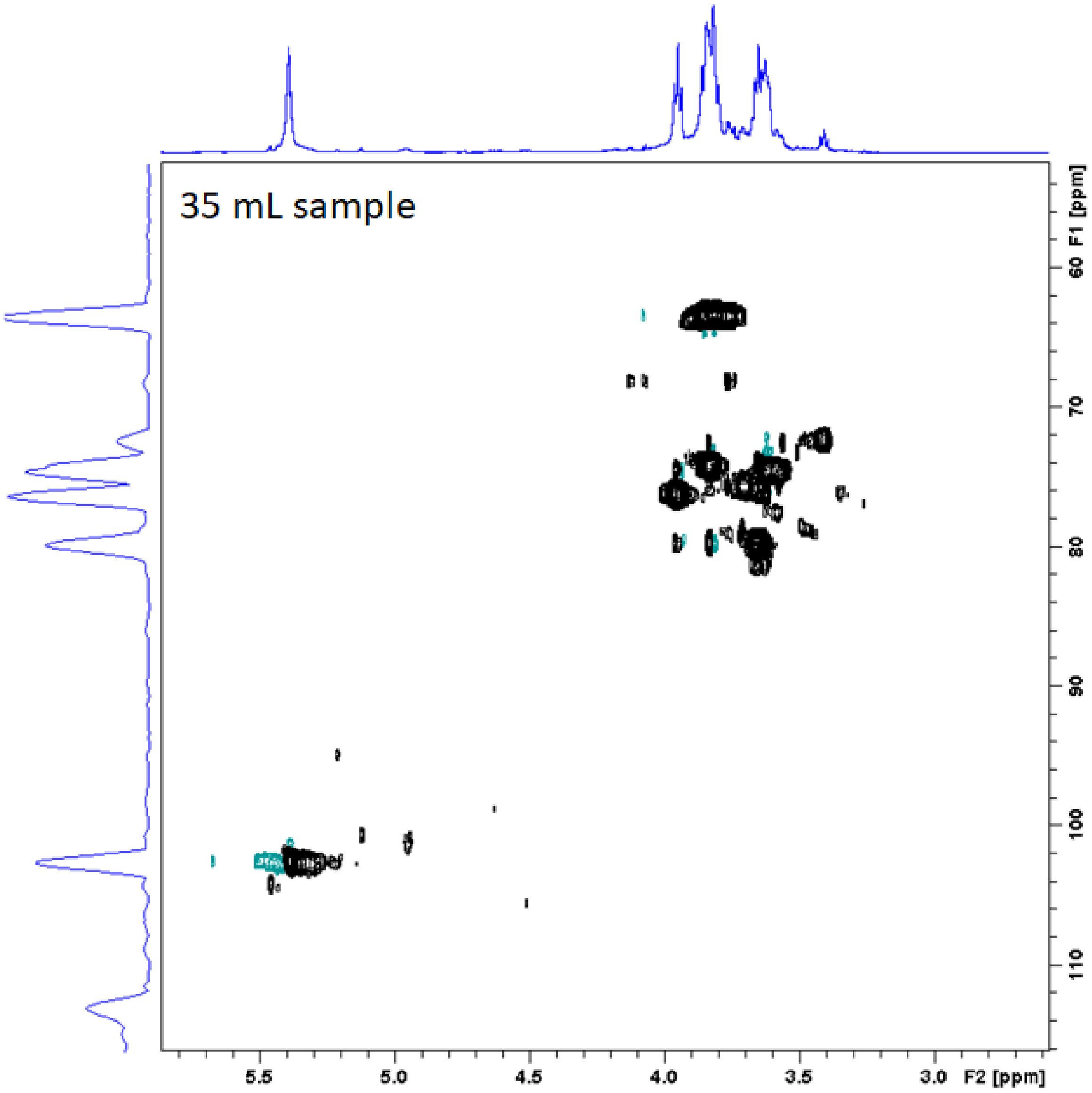

Figure S3. Representative ^{1}H-^{13}C HSQC spectrum for 35 mL sample.
